# Supplementary material for: Are carbon emissions trading and green financial instruments synergistic? -Comprehensive quantitative research based on content analysis
Source: PLoS One. 2024 Mar 7;19(3):e0298601. doi: 10.1371/journal.pone.0298601 (PMC10919601; doi:10.1371/journal.pone.0298601)
Supplement: S1 Data — (ZIP) [file pone.0298601.s001.zip › Data/地级市Z_F工作报告.zip!/║■▒▒/╛ú╓▌╩╨/└┤╫╘╣1⁄2╓┌║┼í╛┬φ┐╦╩2╛▌═°í┐_2013.docx]

各位代表：

　　现在，我代表市人民政府向大会作工作报告，请予审议，并请各位政协委员和列席人员提出意见。

　　一、过去一年工作回顾

　　2012年，是“壮腰工程”扎实开局之年，是荆州经济社会发展全面提升之年。面对复杂多变的外部环境，面对经济下行压力加大的严峻形势，在省委、省政府和市委的正确领导下，在市人大、市政协的监督支持下，我们紧紧依靠全市人民，高举“壮腰工程”大旗，提升精气神，奏响主旋律，较好地完成四届人大二次会议确定的主要目标任务，全市经济社会发展呈现新变化：政策机遇不断汇集，叠加效应充分释放，为荆州振兴营造了强大气场;开放开发势头强劲，固定资产投资突破千亿大关，为荆州振兴打下了坚实基础;干部群众凝心聚力、士气高昂，为荆州振兴注入了持久动力。可以说，荆州发展正处于历史上最好的时期，荆州发展站在了新的起点上。

　　(一)致力保增长，发展态势更加稳健。把产业突破作为经济工作的首要任务，壮大实体经济，加快结构调整，经济总量和质量稳步提升。据初步核算，全市生产总值1196.02亿元、增长11.1%，三次产业比调整为24.5：43.7：31.8，第二产业比重提高0.7个百分点;固定资产投资1042.89亿元、增长35.2%;地方一般预算收入56.76亿元、增长28.1%.

　　工业经济逆势增长。强力推进“工业壮腰”，支柱产业和骨干企业发展加快。规模以上工业增加值434.06亿元、增长16.8%，连续五年超全省平均增幅。规模以上工业企业净增91家、达到804家，实现利税80亿元、增长28%;销售收入过亿元企业新增72家、达到280家，福娃集团、中兴能源突破50亿元。荆州开发区白色家电产业集群纳入省重点扶持;农产品加工业达到700亿元、增长31%.全市新增高新技术企业13家、达到60家，菲利华公司成为国家创新型试点企业;高新技术产业增加值81.88亿元，占全市GDP的6.8%，超额完成省政府考核目标。

　　农业经济持续增长。粮食生产实现“九连增”，达到371.06万吨、增长2.2%，我市荣获“全国粮食生产先进市”称号，监利、洪湖、公安荣获“全国粮食生产先进县”称号。油料总产57.55万吨、增长3.1%，连续四年突破千万担;淡水产品产量115.69万吨、增长7.1%，连续十八年稳居全国第一，中国淡水产品交易中心落户荆州;生猪出栏474.4万头、增长5.1%.监利、洪湖、松滋被纳入全国高标准基本农田示范县。农业产业化市级以上重点龙头企业新增62家、达到265家，农民专业合作社新增702家、达到2202家。“天助”牌食用油荣获“中国驰名商标”，新增“洪湖莲子”等11个“国家地理标志证明商标”。华中农高区成为全省第一个农业高新技术产业开发区。

　　服务业稳步增长。全市实现社会消费品零售总额650.54亿元、增长16.1%;外贸出口9.22亿美元、增长19.8%，高于全省平均增幅10个百分点以上;实际利用外资9020万美元、增长20%.古城人口疏散前期工作启动，大遗址保护工程、“洪湖岸边是家乡”、洈水风景区等重大旅游项目加快推进，洪湖“悦兮半岛”升级为4A景区，楚王车马阵景区对外开放。全市共接待游客1562.6万人次，实现旅游总收入92.6亿元，分别增长29.2%和31.1%.现代物流规划编制完成，成功获批“全国中小商贸流通企业服务体系建设试点城市”，被确定为省级化工、汽车及零部件出口基地。农村金融服务实现全覆盖，交通、平安两家股份制银行入驻荆州，新增银行贷款100.35亿元、增长18.4%，连续五年被评为省“A级信用市州”。

　　(二)致力抓投资，发展后劲更加强劲。把投资作为重要引擎，把项目作为重要抓手，把园区作为重要平台，高强度推进，高密度督办，投资成为“壮腰工程”的最大亮点。

　　园区建设明显提速。抢抓国家级承接产业转移示范区建设机遇，荆州开发区规划面积拓展到209平方公里，“两纵六横”道路及配套建设加快;实行中心城区“四不变、四统一”政策，共建共享国家级开发区的体制机制初步形成。江陵开发区获省政府批准，沿江产业园初见雏形。荆州区临港工业新城、沙市开发区、松滋临港工业园、公安青吉工业园、监利城东工业园、石首金平工业园、洪湖新滩工业园扩容工程全面铺开。全市10个开发区投入建设资金近50亿元。

　　招商引资成效显著。扎实开展驻点招商，成功举办“国际关公文化学高峰论坛暨湖北壮腰工程招商大会”和“省企业界人大代表楚天行”等重大招商活动，全市引进项目773个(其中工业项目593个)，实际到位资金490亿元、增长52.8%，项目数量和到位资金均创历史之最。招商质量明显提高，各县市区引进过亿元工业项目均超过15个，荆州开发区引进10亿元以上工业项目7个;全市新引进投资过10亿元的重大项目46个，其中工业项目30个。项目落地明显加快，新开工工业项目达482个。

　　发展环境不断优化。坚持开展“治庸问责”、政风行风评议活动，下大力减项目、减环节、减时限、减费用，下放行政审批权限138项;工业项目审批时限由156天缩短为60天，“全程代办”机制建立健全，基本做到“零收费、零障碍”，荆州开发区创造了项目落地的“荆州速度”。创新财政资金投入方式，设立全省市州第一支创业(产业)投资引导基金。项目建设用地、水电、天然气等要素需求得到保障。“大部制”改革纵深推进，12家市直部门和单位归并整合。

　　(三)致力管长远，发展基础更加坚实。坚持城乡统筹发展，加快推进城乡一体化进程，一批支撑荆州长远发展的重大基础设施项目取得突破，城乡面貌呈现新变化。

　　综合交通建设步伐加快。“北煤南运”大通道建设拉开序幕。江南高速、洪监高速建设步伐加快，东卷高速、荆松一级公路、武汉城市圈环线洪湖段高速公路、郢城客运枢纽工程开工建设。引江济汉工程顺利推进，荆州组合港“一港十区”建设全面铺开，港航建设开工项目数和进度领跑全省。沙市机场通用航空资格得到恢复。特别可喜的是，汉宜高铁开通营运，极大地提升了荆州在全国、全省的战略地位。

　　城市建设管理水平提升。沙北新区、荆北新区建设稳步推进，明月公园、滨江公园征迁工作基本完成。海子湖生态文化旅游区总体规划获省政府批准，基础设施建设启动。城市经营水平得到提高，中心城区城建投入达到15亿元，县市城区基础设施投入超过30亿元。新一轮“四城同创”活动启动，社区环境等专项整治扎实开展。市区街道三级城管机制得到巩固，城市管理长效机制基本建立，逐步向数字化、精细化、规范化转变。

　　农村基础条件不断改善。统筹城乡规划编制，基本实现建制镇(场)总规和村庄规划全覆盖。监利新沟、洪湖峰口、沙市岑河、松滋刘家场等省级重点乡镇建设加快，白螺镇纳入省级口子镇发展战略。新建新型社区116个、通村公路649公里、户用沼气池1920户，新增农村安全饮水人口32.5万人，植树造林22万亩。水利建设投入17.5亿元，更新改造大型排涝泵站18处，除险加固中小型水库29座，荆江大堤综合整治、荆南四河堤防加固工程可研报告获国家发改委批复。申报实施高产农田土地整治项目42个，建设规模79.2万亩，新增耕地2.83万亩。“福娃模式”成为全省城乡一体化发展的新典型。

(四)致力保民生，发展成果更加普惠。坚持向民生倾心力、倾财力，千方百计办实事、办好事，解决了一大批事关人民群众切身利益的热点、难点问题，民生福祉不断改善。

人民生活水平稳步提高。城镇居民人均可支配收入17010元、增长13.8%，农民人均纯收入8710 元、增长13.7%.“十件实事”圆满完成。新增城镇就业82282 人，荣获“全国创业先进城市”称号。城乡居民社会养老保险实现全覆盖，参保人数249.43万人;城镇职工社会保险参保人数382.98 万人次;征缴社保基金45.16 亿元;企业养老保险及时足额发放，人平月均待遇达到1420 元。保障性住房开工建设23629 套，竣工15045 套，发放租赁补贴3685 万元。社会救助体系进一步健全，城乡低保人数达到33.1 万人，发放救助资金6.3 亿元。

社会管理创新不断加强。公众诉求平台基本建立，社区网格化管理综合服务体系建立健全，市级财政投入资金2600 万元，1221 个网格实现“一格一员”。继续推行信访积案包案制，化解积案328 件，矛盾纠纷大调解机制进一步健全。警务机制创新力度加大，公众安全感和治安、公正执法满意度提升。安全生产形势平稳好转，事故控制指标连续八年保持下降趋势。食品药品安全监管加强。应急处置能力提高。

社会事业全面进步。荣获“省科技创新先进市”称号。新建和改扩建幼儿园29 所，中心城区教育资源整合与学校布局调整启动实施，全面取消义务教育阶段“择校费”，松滋市通过县域义务教育均衡发展省级评估，高等教育“校地合作”加强。文化惠民工程实现全覆盖，全民阅读活动蓬勃开展。成功举办张居正国际学术研讨会。国有文化单位转企改制，湖北长江垄上传媒集团成功组建，荆州日报跻身湖北十大报刊传媒行列。市体育中心开工建设，省运会筹备工作进展顺利。“健康荆州”全民行动启动，血防灭螺1.6 亿平方米，免费救治晚血病人2253 人。严厉查处“两非”案件，出生人口政策符合率达到94.09%.完成节能项目19 个，单位生产总值能耗下降3.6% .完成减排项目59 个，饮用水水源地、危险废物、重金属排放等专项整治取得实效，洪湖生态治理、中心城区内河补水、西干渠整治、农村环境连片整治等工程顺利推进，荆州开发区污水处理达标排江工程建成启用。文明创建活动深入推进，涌现出人民调解员雷体华、“大爱父母”黄宏林夫妇、寒江救人的维族学生麦热达尼等一批先进典型。妇女儿童、老年人和残疾人合法权益得到保护。国防动员、国防教育、人民防空和民兵预备役工作得到加强。审计监督质效提升。统计服务经济发展的能力进一步增强。以整村推进为重点的扶贫开发顺利实施。档案管理信息化建设力度加大。物价、外事侨务、民族宗教、台湾事务、邮政通讯、防震减灾等工作取得新成绩，供销、烟草、盐务、检疫、移民、气象、社科、史志、保密和无线电等工作取得新进步。

(五)致力强气场，发展信心更加坚定。用“壮腰”的目标凝聚人心，用振兴的政策汇聚能量，用大好的机遇鼓舞士气，“荆州信心”明显增强。

政策机遇进一步汇集。“壮腰工程”成为加快荆州发展的重要契机，成为荆州争取合作的重要平台，产生了巨大的集合效应、放大效应。积极争取上级支持，荆州被纳入长江中游城市群、洞庭湖流域生态经济区建设规划。国家老工业基地调整改造正式启动。省政府出台支持承接产业转移示范区建设的政策意见，设立10 亿元“壮腰工程”重大产业发展基金。省委、省政府召开荆州旅游开发现场办公会。与85 个省直部门和单位签订厅市合作协议，未来五年省级投融资平台计划在荆投资超过600 亿元，各金融机构授信总额达到1000 亿元，水利、卫生、林业、移民等民生事业计划投入超过200 亿元。“壮腰工程”汇聚了前所未有的政策机遇，激发了全市人民的高度自觉和自信，形成了强大的“荆州气场”。

发展合力进一步增强。自觉接受人大法律监督和政协民主监督，认真落实人大常委会决议，主动向人大报告工作，向政协通报情况，加强与各民主党派、工商联、无党派人士、人民团体的联系。办理人大代表建议111 件、政协委员提案280 件。民主法制建设进一步加强，依法治市稳步推进，政务公开、新闻发布、政务督查力度加大，依法行政水平不断提高。认真落实党风廉政建设责任制，加大对违法违纪案件的查处力度，廉政建设不断加强。深入开展“创先争优”主题实践活动，形成了人心思齐、人心思上、人心思干的“大合唱”。

各位代表!一年辛苦不平凡，一年变化不寻常。变化令人鼓舞，变化催人奋进，变化来之不易。无论是昨天的成绩，还是今天的变化，都凝聚着全市人民的汗水和智慧，诠释着荆州发展的律动和轨迹，展现出荆州振兴的蓬勃生机和美好前景。在此，我代表市人民政府，向所有参与、关心和支持荆州发展的同志们、朋友们，表示衷心的感谢并致以崇高的敬意!

各位代表!在总结成绩的同时，我们也清醒地看到，荆州发展还存在许多困难和问题：发展不快、发展不优仍是当前荆州最大的实际，由于多种因素影响，地区生产总值、规模以上工业增加值、社会消费品零售总额增幅离预期目标还有一定差距;产业结构调整不快，生产要素供应趋紧，企业生产成本上升，部分企业特别是中小企业经营困难;城乡基础设施建设还有差距，城乡居民收入持续增长难度加大，维护社会稳定压力较大;工作作风转变不够到位，少数政府工作人员不作为、慢作为、不落实等现象依然存在。迎接新的挑战，我们必须更加自觉、更加坚定、更加努力，不断自加压力，改进工作，把荆州的事情办得更好。

二、2013 年的主要工作目标和任务

2013 年，是全面贯彻落实党的十八大精神的开局之年，是实施“壮腰工程”、加快荆州振兴的发力之年，是为全面建成小康社会奠定基础的重要一年。

全面建成小康社会，我们蓄势待发。“壮腰工程”将荆州发展推向了全省的前台，区位、资源、基础优势日益凸显。我们必须立足基础优势，发挥资源优势，做大比较优势，摒弃旧的羁绊，开创新的局面，让荆州经济乘势而上、跨越发展。

全面建成小康社会，我们信心满怀。党的十八大吹响了全面建成小康社会的号角，一系列政策利好令人期待。国家大力实施“中部崛起”战略，规划布局长江中游城市群和洞庭湖流域生态经济区建设，省第十次党代会为荆州发展描绘了宏伟蓝图。我们必须抢抓机遇、把握机遇、用好机遇，把各方面的政策机遇转化为项目资金，转化为助推振兴的强大动力。

全面建成小康社会，我们任重道远。对标小康社会的宏伟目标，荆州在人均生产总值、人均财政收入、城镇化水平等方面都存在较大差距，是制约荆州全面建成小康社会的“短板”。我们必须顺应发展大势，顺应人民期盼，脚踏实地，奋起直追，干好今年，赢得未来。

今年政府工作的总体要求是：认真贯彻落实党的十八大、中省经济工作会议、省第十次党代会和市第四次党代会精神，以科学发展为主题，以转变发展方式为主线，以改革开放创新为动力，以改善民生为根本，坚持“稳中求进、好中求快、竞进提质”，全面实施“壮腰工程”，着力推进“四化同步”发展，奋力打造湖北经济增长“第四极”，为全面建成小康社会打下坚实基础。

今年经济社会发展的主要预期目标是：生产总值增长12% ，固定资产投资增长30%，规模以上工业增加值增长18%，社会消费品零售总额增长15.5%，外贸出口增长15%，实际利用外资增长20%，地方一般预算收入增长15%，居民人均可支配收入增长12%，人口自然增长率控制在6‰以内，居民消费价格涨幅控制在4.5%左右，单位生产总值综合能耗下降3.5%，化学需氧量、二氧化硫、氮氧化物、氨氮排放总量分别下降3%、4%、2%和4%.

围绕上述目标，我们将着力抓好以下五个方面的工作：

(一)壮大产业，增强支撑力。产业突破是振兴荆州的战略之举。坚持“产业第一”、“实体优先”，加快产业规模扩张、结构调整和转型升级步伐，增优势、创特色、提质效，促进三次产业协调发展。

打造工业主导优势。坚持把新型工业化作为“壮腰工程”的第一支撑力，全力以赴大办工业、办大工业。加快集群发展，打造产业优势。着力推进“千亿和五百亿产业发展计划”，实行“一个产业、一个规划、一名领导、一个专班、一套政策、一抓到底”的工作机制，发展壮大农产品加工、医药化工、装备制造、电子信息、轻工建材、纺织服装等产业，力争把农产品加工业打造成为首个千亿级产业;瞄准新材料、新能源和生物制药等产业，促进战略性新兴产业规模发展，抢占未来发展制高点;强化与中、省能源企业对接，谋划发展煤电、煤化工产业;加强产业配套，促进集群发展，力争每个县市区都有1至2 个产销50 亿元以上的产业集群。做大市场主体，做强企业优势。着力推进“百亿级企业培育计划”，落实减税政策，保障要素供应，扶持美的、恒隆、法雷奥、沙隆达、四机厂、白云边、钢管厂、楚源、凯乐、拍马等企业向50 亿元销售收入目标迈进;鼓励企业引进战略投资者，增资扩股、发行债券、兼并重组、争取上市，进一步做大做强;着力推进“中小企业成长工程”和“市场主体增量行动”，力争规模以上工业企业净增100 家。提升产品质量，打造品牌优势。着力推进“质量兴市战略”和“商标战略”，引导企业以质量求效益，以品牌赢市场，年内新增2 至3个中国驰名商标。

增强农业基础优势。更加自觉地把“三农”工作放在重中之重的位置，把城乡一体化作为解决“三农”问题的根本途径，夯实农业基础，促进农民增收。大力推进农业产业化。推广“福娃模式”，完善农企利益联结机制，发展壮大优质水稻、名优水产、双低油菜、优质畜禽、特色蔬菜等优势产业板块，支持福娃、中纺油脂、大明水产、德炎水产、监利鸿翔、松滋襄大、江陵宏凯、荆州雨润等企业加快发展;以华中农高区为龙头、以国有农场为基础，加快现代农业示范区建设;引导“非农”资本投向农业，培育新型经营主体，扶持发展联户经营、专业大户和家庭农场。大力推进农业科技化。围绕主导品种和主推技术，探索科技人员直接到户、良种良法直接到田、技术要领直接到人的农技推广体系和机制，切实解决农技推广“最后一公里”问题;推进标准化生产，实行农产品质量安全市场准入制度;推进生产装备和设施现代化，力争主要农作物机械化综合作业水平达到67%;积极争创“三品一标”，力争培植1 至2 个在全国有影响的农业品牌。大力推进农业服务社会化。扶持发展多种形式的新型农民合作组织和多元服务主体，力争农民专业合作社新增300 家以上;有序推进土地流转，发展农业规模经营;合理规划村庄布局，加快推进新农村建设，努力改善农村生产生活条件。

培育文化旅游支柱产业。按照“大手笔、大投资、大制作、大产业”的思路，做历史文章，兴文化产业，树旅游品牌，让文化旅游资源“活”起来，让文化软实力变成硬支撑。加大资源整合力度，做实投资平台，做大市场主体，加快建设以楚文化为代表的大遗址保护示范区，以关公文化为代表的荆州古城景区，以生态文化为代表的海子湖生态文化旅游区和洪湖、洈水风景区。充分发掘历史文化底蕴，开发富有荆楚特色的文化旅游产品。加快旅游设施标准化建设，完善“吃住行、游购娱”旅游要素。加大市场营销力度，发展区域性旅游共同体和城市旅游联盟，力争实现旅游收入120亿元、增长30%.支持新闻传媒、数字动漫、文化创意等产业发展，加快电子商务、信息咨询、服务外包等现代服务业发展，促进文旅融合、城旅融合，文化旅游与其它服务业的融合发展。

(二)扩大投资，增强带动力。牢牢把握荆州现阶段发展特点和规律，把投资作为扩内需、稳增长、促调整的现实举措，更好地发挥投资在跨越发展中的“龙头”作用。

抓好承接平台建设。加快国家级承接产业转移核心区和集中区建设，提升园区承载能力。以国家级和省级开发区为重点，以提质扩容、完善功能为核心，力争荆州开发区新建园区10 平方公里以上，各县市区新建园区5 平方公里以上。支持荆州开发区用“国家队”标准、“产城融合”理念，加强规划建设，创新经营管理，尽快建成城市新城和产业集聚区、创新示范区、发展率先区。

支持华中农高区以“四化同步”为动力，探索市场化运作模式，努力打造科研、生产、加工、物流综合优势。支持县域特色园区建设，重点抓好荆州区四机工业园和拍马工业园、沙市岑河针纺工业园、江陵煤电路港产业新城、公安循环造纸工业园、松滋白云边工业园、石首张城垸高新工业园、监利服装工业园、洪湖府场石化装备工业园建设。加强与上海嘉定、江苏昆山等沿海发达城市的合作，积极发展“飞地经济”。

提高招商引资质量和效益。抓住沿海产业梯度转移机遇，发挥荆州资源、产业等优势，加强与产业输出地的对接合作。坚持不懈抓好驻点招商，强化产业招商，围绕资源招大商、围绕支柱抓龙头、围绕产业抓配套，提升产业规模和素质。注重信息筛选，持续跟踪行业龙头和上市企业投资动态，将招商“触角”延伸到民资最富集、产业最密集的区域，确保“走出去、扎住根、引进来”。注重以商招商，充分发挥商会、行业协会等社会组织的作用。注重统筹协调，整合招商资源，形成招商合力。实施重大项目会审、服务协调联席会议和考核奖惩制度，力争招商引资到位资金735 亿元、增长50%，省外资金占比达80%以上。

着力抓好项目建设。认真研究国家产业政策和区域发展战略，策划、跟踪、争取一批影响全局、支撑长远发展的大项目。继续强力推进项目建设，加强跟踪服务，建立健全现场办公、并行作业、联动服务机制，促进项目早投产、早见效。产业方面：加快推进长江镍业、同洲电子、伟特电子、恒宇精密薄板、拍马烟用卡纸、耀能光伏、美帆金属、永发纸业、中哈能源、秦楚纸业、楚源扩能、上信服装、千点食品等重大工业项目建设，抓好古城人口疏散和保护利用、楚王车马阵、关公文化园、垄上行文化产业园、“洪湖岸边是家乡”、九老仙都景区等文化旅游项目建设。交通方面：加快推进“北煤南运”铁路、江南高速、洪监高速、东卷高速、武汉城市圈环线洪湖段高速、荆松一级公路、引江济汉工程、荆州组合港等在建项目建设，开工建设潜石高速、江北高速、沙公高速、中心城区318 国道改建、旅游大通道等项目，抓紧做好沙市机场迁建和“北煤南运”江陵煤炭储运基地前期工作;加快物流园区建设，带动生产性、生活性服务业发展。水利方面：加快推进荆江大堤综合整治、荆南四河堤防加固、四湖流域综合治理、洪湖分蓄洪区东分块、荆江河势控制、水库整险加固、大型灌溉和泵站改造等项目建设，提高防洪抗旱减灾能力。

(三)创新驱动，增强内生力。紧紧抓住新一轮产业升级、结构调整的战略机遇，利用市场“倒逼”机制，加大改革创新力度，为荆州转型发展提供不竭动力。

提升自主创新能力。统筹抓好科技创新和产业转型，突出企业主体，注重协同创新，促进“产学研”结合。发挥在荆高校、科研院所资源优势，探索建立产业技术创新战略联盟。扶持荆州开发区国家级创业中心、华中农高区农科园和省级以上企业工程技术中心、公共检测平台建设，促进科技成果产业化。推进信息化与工业化、农业现代化深度融合，加强信息技术在产品设计、生产、管理、营销等环节的集成应用，提升中小企业信息化水平。积极争取国家、省专项资金，引导企业加大科研技改投入，力争技改项目达到100个以上。继续推进“人才优先”发展战略，实施“金凤人才”计划，加大引智力度，培育和造就一批发展急需的领军型、创新型和技能型人才队伍。

深化体制机制创新。稳步推进财税体制改革，做好“营改增”试点工作，探索财政专项资金竞争性分配改革。强化国有资产监管，推进与央企、省企对接合作，支持中国电子集团重组南机总厂。鼓励和引导非公有制经济发展，消除有形和无形壁垒，放宽市场准入，规范市场秩序。积极引进股份制银行，规范发展金融保险、小额贷款、各类担保公司和证券公司，规范投融资平台建设，降低实体经济融资成本。深化农村综合改革，实行农村集体资金、资产、资源统一管理，推进“城中村”、“城郊村”、“园中村”集体资产产权制度创新。稳步推进“大部制”改革和事业单位分类改革。

推动发展环境创新。营造尊商、亲商、护商、利商的文化氛围，用良好的人文环境呵护投资者、善待创业者、优待企业家。进一步精减审批事项，完善批管分离，优化审批流程，落实工业项目“全程代办制”，推广荆州开发区服务企业“秘书制”经验，全面提高行政服务效能。千方百计破解项目建设用地、用电、用水、用气、用工等方面的瓶颈制约，严厉打击强买强卖、阻挠施工等违法行为。更加重视“草根经济”，为本土企业成长壮大营造更宽松的环境。加强信用环境建设，在招商引资中慎重承诺、痛快兑现，维护政府诚信形象。继续争创省“A 级金融信用市”，巩固“最佳金融县市”全覆盖成果。

(四)拓展城市，增强承载力。遵循城市发展规律，顺应人民群众期待，加快推进城乡一体化发展，建设“美丽荆州”、“幸福家园”，让更多的市民在城市安居乐业，让更多的农民到城市安家落户。

加快中心城区建设步伐。转变城市发展方式，用先进的理念规划城市、引领发展，促进经济社会发展、土地利用、城市总规“三规统一”。推进城市有序扩张，加快沙北新区、荆北新区、开发区商务新区、海子湖新区、李埠临港新区建设，着手启动江南新区规划建设，积极发展郢城、岑河、关沮、锣场等城市外围组团，拉开城市构架。用市场化的手段筹集资金，建设一批城市综合体、产业地产项目，促进产业与城市互动发展。加快推进行政中心、文体中心建设，改造升级城市出入口和荆州大道、沿江大道、北湖路、凤凰路等主次干道，创建荆南路、江津东路和塔桥路等省级示范路，开展沙市区荆沙村、荆州区城南片区整村改造试点，抓好城市公交、公园、公厕设施和生态林、道路绿化带、滨水生态走廊建设，建成明月公园、滨江公园。在注重城市地上建设的同时，更加注重地下管网建设，全面提升城市功能。

加速城镇化进程。抓紧编制县市城区、集镇、新型农村社区规划，实现控制性详细规划全覆盖。加强城乡规划监管，严控严查违法违章建设。推动工业化、城镇化良性互动，以县域为重点，以乡镇为载体，以产业为支撑，推进人口向城镇集聚。加快县市城区“中等化”步伐，重点抓好省级重点镇、特色镇建设，构筑以中心城区为龙头、县市城区为支撑、重点镇为节点的沿江沿路城镇带。推进城乡融合发展，以工促农，以城带乡，加快城市公共服务基础设施向农村延伸，统筹解决户籍、就业、子女就学、公共卫生、住房租赁、社会保障等方面的实际问题，促进农业转移人口市民化，充分释放城镇化扩内需、促增长的巨大潜能。

提高城市管理水平。扎实开展中心城区新一轮“四城同创”活动，带动县市创建。深化管理创新，推进城管、交管、治安、工商等方面的信息互通和工作联动，构建“大城管”机制，提高城市管理智能化、规范化、长效化水平。深入开展社区环境、农贸市场、背街小巷、交通秩序等专项整治，抓好国家园林城市复查迎检和国家卫生城市创建申报工作。继续实施绿化、美化、净化、亮化工程，把管理服务的触角向背街小巷、居民小区拓展延伸。依靠群众管理城市，充分发挥好志愿者作用，努力把深厚的文化底蕴内化为广大市民的文明素养，在细节管理中体现人文关怀，在潜移默化中传播文明种子。

(五)改善民生，增强凝聚力。更加关注人民生活质量、幸福指数，切实做好保障民生的制度性安排，努力让发展成果体现在民生福祉上，让老百姓看到变化、得到实惠。

提高公共财政保障水平。强化税收征管，优化税种结构，不断扩大增量、做大总量、提高质量。管好用好重大产业发展基金和政府引导基金，充分发挥财政资金杠杆作用，扶持重点企业特别是战略性新兴产业发展，涵养培植新税源。从严控制一般性支出，压缩“三公”经费，逐步实现由吃饭型向发展型财政转变。着力保障强农惠农、创业就业、社会保障、社会事业、扶贫开发等民生投入。多渠道增加居民收入，规范收入分配秩序，努力实现更有“福利”、更有“温度”的增长。

提高公共服务水平。开展国家级创业型城市动态创建工作，扶持创业带动就业。加强社保扩面征缴、转移衔接工作，增强公平性，适应流动性，促进可持续性。继续实施学前教育三年行动计划，新建和改扩建幼儿园40 所;优化城乡中小学布局，新建学校6 所，改扩建学校92 所，完成标准化建设学校222 所，争取2 至3 个县(市、区)通过省级县域义务教育均衡发展示范县评估;加快荆州中学、沙市中学迁建;加强技能型人才培养，创建国家级示范中职学校1 至2所、省级示范中职学校1 所;支持长江大学等在荆高校加快发展。加快推进图书馆、档案馆、文化馆建设，开展全民阅读活动。抓好文物保护、非物质文化遗产保护与传承。新建社会化养老服务机构40 所，实施中心城区殡仪馆搬迁重建。推进依法治市，扎实抓好“六五”普法工作。抓好国防动员、民兵预备役、人民防空工作，支持驻荆部队建设。加强社会主义精神文明建设，推进文明创建活动，践行社会主义核心价值观。适时启动价格调节机制，实行社保与物价“挂钩”联动，确保低收入群体生活质量不因物价上涨而下降。加强统计基础工作，发挥信息、咨询和监督作用。统筹抓好专项扶贫、行业扶贫、社会扶贫，构建“大扶贫”格局。抓好外事侨务、台湾事务、民族宗教、防震减灾、移民、盐务、邮政、供销、气象、史志、保密、无线电等工作，维护好残疾人和妇女、儿童权益。

加强和创新社会管理。依托网格化管理综合服务平台，推进资源集成、力量下沉和功能拓展，加快建立源头治理、动态管理、应急处置相结合的社会管理机制。推进“三网”融合，抓好信息技术在社会管理中的应用，积极打造基于云计算、大数据的智慧城市。高度重视信访维稳工作，增强运用法治思维和法治方法化解矛盾纠纷的能力，引导群众通过合法渠道、理性方式表达利益诉求。强化生产安全、消防安全监管，确保不发生重特大安全事故。构建社会治安防控体系，完善城市治安、道路交通安全视频监控设施，严厉打击各类违法犯罪活动，努力建设“平安荆州”。

提高人民健康水平。深入开展“健康荆州”全民行动，组织好“万人洁城”活动、第三轮“三万”活动，整治城乡环境卫生，倡导健康文明生活方式。继续开展医疗质量“荆楚行”、“平安医院”创建行动，保障医疗质量安全。启动新一轮医疗机构服务体系建设，实施新农合市级统筹，建立健全大病保障和救助机制。按照“三步走”战略要求，实现血吸虫病传播控制达标目标。健全食品药品安全检验检测体系，加强食品药品全过程、可追溯监管，努力让人民群众买得放心、吃得安心。加快建设和改造16个体育场馆，广泛开展全民健身运动。切实稳定低生育水平，提高出生人口素质。推进资源节约集约利用，严格按照投资强度核定项目用地规模。强化企业污染、机动车尾气排放监管，扎实开展农村面源污染治理，加快洪湖、长湖生态修复，不断提高生态文明建设水平。

认真办理十件实事。一是新增就业70000 人，扶持创业5000 人，带动就业20000人，创业培训5000 人，发放小额担保贷款2亿元。二是开工建设保障性住房16840 套，竣工12000 套，新增租赁补贴1350 户。三是为困难群众和农民工办理法律援助案件2000 件，办理法律援助事项20000 件以上。四是解决农村30.62 万人的饮水安全问题。五是实施85 岁以上老年人高龄补贴。六是免费培训农民2 万人，新建村级综合服务社200 家。七是免费为2.6 万对农村夫妇提供孕前优生健康检查，为100 名贫困残疾人安装假肢，为200 名贫困白内障患者实施复明手术，为1000 名贫困精神残疾人提供基本控制药物，为200 名0 至6岁贫困残疾儿童实施抢救性康复，为100名中心城区贫困残疾人实施家居无障碍改造，为500 名0 至14 岁残疾儿童和残疾人家庭子女实施集善残疾儿童助养项目。八是更新和新增城市公交车100 台，新建公交枢纽场站1 个，开办残疾人免费公交卡。九是为2.8 万名普通高中家庭经济困难学生发放助学金3600 万元。十是为37 个社区文化活动室配置标准文化设备，提升112个公共电子阅览室设备水平。对上述事项，我们将一诺千金，说到做到，努力把实事办实、把好事办好。

三、加强政府自身建设

“空谈误国，实干兴邦”。加快荆州振兴，全面建成小康社会，是一项历史的重任，更是一个奋斗的过程。面对新形势、新要求，我们必须认真落实中央“八项规定”，更加自觉地转变作风，“实”字当头，“干”字为先，努力打造创新型、服务型、责任型、廉洁型政府。

(一)解放思想，在“求实”上下功夫。振兴之路无坦途，解放思想无止境。荆州面临的机遇前所未有，我们肩负的重任前所未有。开启荆州振兴新征程，需要我们有高远的理想、开阔的视野，更要有求实的勇气、开拓的精神。要勇于担责，直面矛盾不回避，应对挑战不畏惧，着力解决改革发展中的深层次、根本性问题。敢于创新，大胆突破条条框框、惯性思维和体制束缚，善于利用改革的办法、市场的办法解决前进中的问题。求真务实，不好高骛远，不急功近利，把握发展大势，驾驭发展大局，结合荆州实际，力求发展目标切实可行，发展举措管用实用，发展速度实实在在，发展成效经得起历史评判和人民考量。

(二)真抓实干，在“踏实”上下功夫。深入调查研究，广泛听取意见，用心谋事，用力做事，努力把困难估计得更充分，把事情考虑得更周全，把工作做得更细致，做到决策“心中有数、胸有成竹”，做到工作“踏石有印、抓铁留痕”。心忧发展之急、民生之急，倾注心血，付出真情，为基层群众多办实事、好事。认真履职，勤奋工作，把心思用在加快发展和改善民生上，努力以广大干部的“辛苦指数”换取人民群众的“幸福指数”。咬定青山不放松，不达目的不罢休，把更多的时间和精力用在“四化同步”、改革开放、改善民生等管全局、打基础、利长远的工作上，用在招商引资、城市建设管理、服务企业和群众等实际工作上，防止抓一阵子、热闹走过场，定了的事坚决一以贯之地抓下去。

(三)从严治政，在“务实”上下功夫。加强同人民群众的血肉联系，真诚聆听来自基层的声音，多到困难和矛盾集中、群众意见多的地方去，认真解决实际问题，化解突出矛盾。坚决反对形式主义、表面文章，不搞虚假政绩，不搞形象工程，做到不扰民、不损民。严格执行“三短一简”规定，提高行政服务效能。牢固树立过紧日子的思想，厉行勤俭节约，反对铺张浪费。严格落实党风廉政建设“一岗双责”制度，认真落实工程建设招投标、国有资产出让招拍挂、政府集中采购和政务公开、新闻发布等制度，强化财政监督、审计监督，努力从源头上预防和遏制腐败，确保政务规范、政纪严明、政府清廉。

(四)强化执行，在“落实”上下功夫。坚持把依法行政贯穿政府工作全过程，做到规范执法、公正执法、文明执法。加强民主法制建设，自觉接受人大法律监督、政协民主监督、媒体监督和社会监督，办好办实人大代表建议、政协委员提案。虚心听取民主党派、工商联和无党派人士建言献策，及时把真知灼见转化为政府决策。坚持把“治庸问责”作为提能善政的重要手段，下大力整治“中梗阻”、慢作为、乱作为等问题。积极构建“落实链条”，推进工作项目化、项目方案化、方案具体化，对重大事项、重点项目明确责任人，明晰时间表，一项一项抓推进，一环一环抓落实，保持硬作风，传递“正能量”，树立新形象。

各位代表!有梦想才有未来，有行动才有希望。让我们在市委的坚强领导和市人大、市政协的监督支持下，心怀振兴梦想，凝聚各方力量，大兴实干之风，认真办好每件事，扎实干好每一年，为打造湖北经济增长“第四极”、全面建成小康社会而不懈奋斗！
